# Pan-cancer molecular tumor board experience with biomarker-driven precision immunotherapy

**DOI:** 10.1038/s41698-022-00309-0

**Published:** 2022-09-22

**Authors:** Bryan H. Louie, Shumei Kato, Ki Hwan Kim, Hyo Jeong Lim, Ryosuke Okamura, Ramez N. Eskander, Gregory Botta, Hitendra Patel, Suzanna Lee, Scott M. Lippman, Jason K. Sicklick, Razelle Kurzrock

**Affiliations:** 1 Center for Personalized Cancer Therapy and Division of Hematology and Oncology, Department of Medicine, UC San Diego Moores Cancer Center, La Jolla, CA USA; 2 Division of Hematology and Medical Oncology, Department of Internal Medicine, Seoul National University Boramae Medical Center, Seoul, Republic of Korea; 3 Department of Internal Medicine, Veterans Health Service Medical Center, Seoul, Republic of Korea; 4 Department of Surgery, Kyoto University Hospital, Kyoto, Japan; 5 Center for Personalized Cancer Therapy and Division of Gynecologic Oncology, Department of Obstetrics, Gynecology and Reproductive Sciences, UC San Diego Moores Cancer Center, La Jolla, CA USA; 6 Division of Surgical Oncology, Department of Surgery, UC San Diego Health Sciences, San Diego, CA USA; 7 WIN Consortium for Precision Medicine, Paris, France; 8 Medical College of Wisconsin Cancer Center and Genomic Sciences and Precision Medicine Center, Milwaukee, WI USA; 9 University of Nebraska, Omaha, NE USA

**Keywords:** Tumour biomarkers, Targeted therapies, Cancer immunotherapy

## Abstract

Despite remarkable responses to immune checkpoint blockade (ICB) in some advanced cancers, most patients do not benefit, perhaps due to the complexity of tumor/immune/genome interactions. We implemented a multidisciplinary Molecular Tumor Board (MTB) that reviewed multi-omic cancer characteristics to develop N-of-One therapies for patients in the pan-cancer, advanced, refractory setting. This study evaluates the experience of 80 patients who were presented to the MTB and received a treatment regimen that included ICB. Overall, 60/80 patients (75%) who received ICB following MTB discussion had a high degree of matching between tumor molecular characteristics, including ICB biomarkers (reflected by a high Matching Score (≥50%)) and therapy administered. Patients with high versus low Matching Score experienced significantly longer median progression-free survival (6.4 vs. 3.0 months; *p* = 0.011) and median overall survival (15.3 vs. 4.7 months; *p* = 0.014) and higher clinical benefit rates (stable disease ≥6 months/partial response/complete response) (53% vs. 21%, *p* = 0.019). Although most patients (52/80 (65%)) received a personalized combination therapy (e.g., targeted, hormonal, chemotherapy, or a second immunotherapy agent), administering >1 drug was not associated with outcome. Only degree of matching and age, but no other variables, including individual biomarkers (e.g., microsatellite status, tumor mutational burden, or PD-L1 status), were independently correlated with outcome. In the pan-cancer setting, the MTB facilitated a precision medicine strategy to match therapeutic regimens that included ICB alone or combined with matched targeted drugs to patients with advanced malignancy, which was associated with improved clinical outcomes.

## Introduction

Immunotherapy has been a transformative development in the treatment of cancer, showing remarkable responses in a subset of patients with many different cancer types, even those with advanced disease^1^. Multiple immune checkpoint inhibitors such as programmed death ligand 1 (PD-L1) or PD1 inhibitors and cytotoxic T lymphocyte associated protein 4 (CTLA-4) inhibitors have been approved, with a broad range of efficacy^2^. However, despite remarkable responses to these agents in some individuals, it is estimated that only ~15–20% of patients with most cancer types exhibit treatment response to immunotherapy, leaving a large majority of patients without clinical improvement^3^.

Treatment resistance to immunotherapy is a complex process involving genes, metabolism, inflammation, and other tumor characteristics that impact response^4,5^. However, recent studies have also identified a number of specific tumor biomarkers whose presence may predict better outcomes with immunotherapy agents^6,7^. For example, tumor microsatellite instability (MSI) and/or deficiency in mismatch repair genes are known to be positive predictors of treatment response to anti-PD-L1/PD-1 therapy across many cancer types^8^. Tumor mutational burden (TMB) has recently been approved by the United States Food and Drug Administration (FDA) as a biomarker for response to pembrolizumab in all solid tumors with TMB ≥ 10 mutations/megabase^9^. There are also numerous genomic characteristics that have been associated with treatment response or resistance to immunotherapy agents^10,11^.

Despite advances in identifying immunotherapy biomarkers, their utility is often limited by complex tumor heterogeneity, especially when multiple genomic aberrations and/or biomarkers cannot be addressed by a single immunotherapy agent or targeted drug. In the field of targeted therapeutics, several studies suggest that the ideal strategy for treatment is a personalized, combinatorial approach in order to maximize matching of drugs to molecular alterations, including immunotherapy biomarkers^12–14^.

To facilitate a clinical precision medicine strategy, we formed a Molecular Tumor Board (MTB)^14–17^. This multidisciplinary team of clinicians and scientists, functions by integrating a broad review of each patient’s unique characteristics, including molecular profiling, pathology, imaging, and clinical history, in order to develop an N-of-One treatment plan recommended for each cancer patient. In this current study, we present 80 patients with multiple types of advanced stage cancer who were presented to the University of California San Diego, Center for Personalized Cancer Therapy Molecular Tumor Board, and were subsequently treated with a drug regimen that included ≥1 immunotherapy (checkpoint inhibitor) agent. Our current study suggests that unique matching of immunotherapy drugs to tumor alterations and biomarkers is associated with improved clinical outcomes.

## Results

### Patient characteristics

**Fig. 1:**
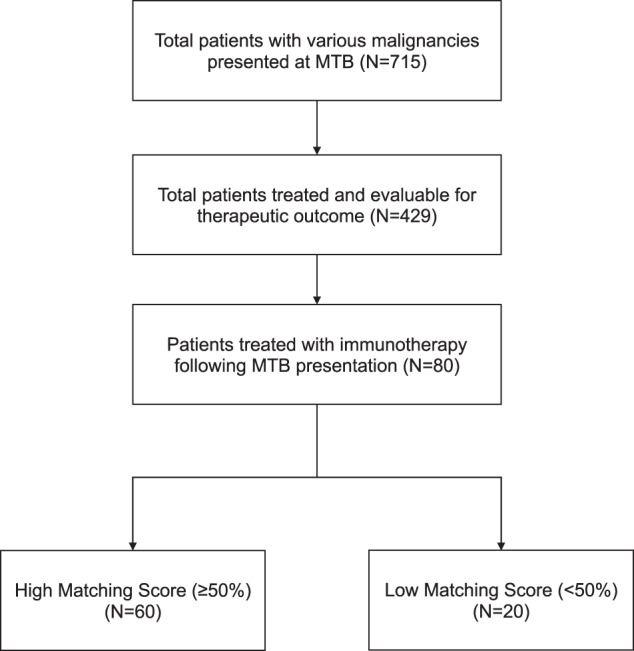
Consort diagram of patients who presented to the face-to-face Molecular Tumor Board (MTB). Of the total 715 patients with various malignancies that presented to the MTB, there were 80 patients that were treated with immune checkpoint inhibitors and assessable for therapeutic outcome.

Of 715 total patients with various malignancies that were presented in the face-to-face MTBs, 80 individuals were treated with immune checkpoint inhibitors and assessable for therapeutic clinical outcome (Fig. 1). Among these 80 patients, the median age was 63 years (range: 13–88). Forty-four patients (55%) were women, and 36 patients (45%) were men. All patients had advanced or metastatic disease and 34 patients (43%) were treated with ≥3 prior lines of therapy. The most common diagnosis was gastroesophageal cancer (15% [12/80]), followed by gynecologic cancer (13.8% [11/80]), colorectal cancer (12.5% [10/80]), hematologic malignancies (10% [8/80]), and pancreatic cancer (6.3% [5/80]) (Table 1). Fifty-two patients (65%) received more than one drug in their immunotherapy regimen and, for the most part, second or third agents included targeted compounds (Supplementary Table 2). Of the 80 patients in this study, 17 (21%) received all drugs recommended by the MTB, 45 (56%) received at least one but not all of the drugs recommended by the MTB, and the remaining 18 (23%) received a physician’s choice regimen, which did not include any drugs recommended by the MTB.

**Table 1:** Baseline demographics of patients presented at the Molecular Tumor Board (MTB) and subsequently treated with immune checkpoint inhibitors (*N* = 80).

Total patients treated with immunotherapy (*N* = 80)	
Period	December 2012–September 2018
Median age at MTB (years) (range)	63 (13–88)
Sex, *N* (%)	Female, 44 (55%); Male, 36 (45%)
Diagnosis	*N*, patients (%)
Gastroesophageal cancer	12 (15%)
Gynecologic cancer	11 (13.8%)
Colorectal cancer	10 (12.5%)
Hematologic malignancies	8 (10%)
Pancreatic cancer	5 (6.3%)
Sarcoma	5 (6.3%)
Breast cancer	4 (5%)
Head and neck cancer	4 (5%)
CNS malignancy	4 (5%)
Biliary cancer	3 (3.8%)
Bladder/ureter cancer	3 (3.8%)
Hepatocellular carcinoma	2 (2.5%)
Other GI malignancies	1 (1.3%)
Other malignancies^a^	8 (10%)
Number of drugs given after MTB^b^	
>1	52 (65%)
1	28 (35%)

Abbreviations: *CNS* central nervous system; *GI* gastrointestinal.

^a^Other malignancies include squamous cell carcinoma (*N* = 2, penile, anal), carcinoma of unknown origin (*N* = 2), basaloid carcinoma (*N* = 1), carcinosarcoma of the endometrium (*N* = 1), cutaneous melanoma (*N* = 1), peritoneal cancer (*N* = 1).

^b^See Supplemental Table 2 for drugs given.

### High versus low degrees of matching of treatment to molecular alterations was associated with longer progression-free and overall survival

Among 80 patients whose treatment regimen included an immune checkpoint inhibitor following MTB presentation, 60 patients received treatment with a high Matching Score (≥50%) (reflecting a greater match of molecular alterations in tumors to therapy given, either because the tumor had specific biomarkers considered by the MTB as a match to immunotherapy [see “Methods” and Supplementary Table 3] and/or because the tumor also had targetable genomic alterations and the patient was given a combination regimen that included a matched targeted agent); the remaining 20 patients received treatment with a low Matching Score (<50%) (Fig. 1).

**Fig. 2:**
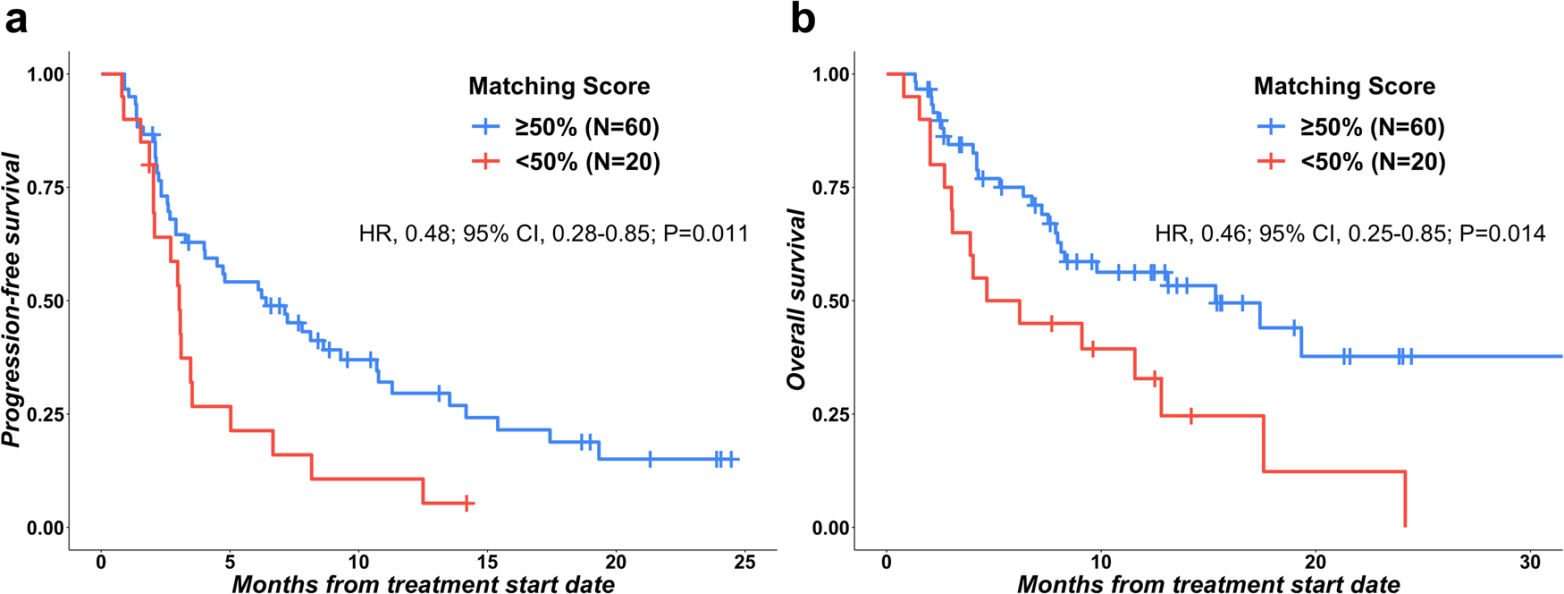
Progression-free survival and overall survival based on Matching Score (*N* = 80). **a** Progression-free survival (PFS) was significantly longer in patients with high (≥50%) Matching Score versus low (<50%) Matching Score (Hazard Ratio (HR), 0.48; 95% CI, 0.28–0.85; *P* = 0.011, univariate Cox regression) (*N* = 80). Median PFS: High (≥50%) Matching Score, 6.4 months (95% CI: 3.3–9.5); Low (<50%) Matching Score, 3.0 months (95% CI: 2.5–3.6). **b** Overall survival (OS) was significantly longer in patients with high (≥50%) Matching Score versus low (<50%) Matching Score (Hazard Ratio (HR), 0.46; 95% CI, 0.25–0.85; *P* = 0.014, univariate Cox regression). Median OS: High (≥50%) Matching Score, 15.3 months (95% CI: 6.07–24.6); Low (<50%) Matching Score, 4.7 months (95% CI: 0.0–9.4).

Patients with high degrees of matching of treatment to cognate alterations (i.e., high Matching Score (≥50%)) experienced significantly longer progression-free survival (PFS) (HR, 0.48; 95% CI, 0.28–0.85; *p* = 0.011) and overall survival (OS) (HR, 0.46; 95% CI, 0.25–0.85; *p* = 0.014) when compared to patients with low degrees of matching (Matching Score, <50%) both by univariate analysis (Fig. 2a, b). The association between Matching Score, PFS, and OS maintained a trend towards statistical significance with multivariate analysis: PFS (HR, 0.58; 95% CI 0.33–1.03; *p* = 0.061), OS (HR, 0.54; 95% CI, 0.28–1.03; *p* = 0.062); no other factors were selected as significant in multivariable analysis (Table 2).

**Table 2:** Association between patient and treatment characteristics, PFS and OS (*N* = 80).

Characteristics		PFS						OS				
				Univariate		Multivariate^b^			Univariate		Multivariate^b^	
		*N*	Median (months) (95% CI)	HR (95% CI)	*P*-value	HR (95% CI)	*P*-value	Median (months) (95% CI)	HR (95% CI)	*P*-value	HR (95% CI)	*P*-value
Age, years	≥63	40	7.80 (5.53– 10.07)	0.61 (0.37–1.01)	0.054	0.70 (0.42–1.16)	0.166	17.40 (7.71–27.09)	0.51 (0.28–0.95)	0.034	0.60 (0.31–1.15)	0.123
	<63	40	2.67 (1.81–3.52)	–	–	–	–	7.53 (3.24–11.82)	–	–	–	–
Sex	Male	36	3.07 (1.24–4.89)	1.44 (0.87–2.38)	0.152	–	–	8.13 (5.31–10.95)	1.48 (0.81–2.71)	0.199	–	–
	Female	44	6.40 (2.44–10.37)	–	–	–	–	17.40 (9.96–24.84)	–		–	–
Matching score (%)	≥50%	60	6.40 (3.30–9.50)	0.48 (0.28–0.85)	0.011	0.58 (0.33–1.03)	0.061	15.33 (6.07–24.59)	0.46 (0.25–0.85)	0.014	0.54 (0.28–1.03)	0.062
	<50%	20	3.03 (2.52–3.55)	–		–	–	4.67 (0.00–9.42)	–	–	–	–
GI malignancies	Yes	28	3.03 (2.08–3.99)	0.93 (0.54–1.58)	0.781	–	–	8.13 (0.00–18.25)	1.15 (0.61–2.18)	0.672	–	–
	No	52	5.03 (2.54–7.53)	–	–	–	–	13.10 (6.00–20.20)	–	–	–	–
Number of prior lines of therapy	≥3	34	4.03 (0.00–8.25)	0.84 (0.51–1.40)	0.511	–	–	17.40 (5.07–29.73)	0.75 (0.40–1.40)	0.366	–	–
	<3	46	4.73 (2.25–7.22)	–	–	–	-–	9.80 (3.22–16.38)	–	–	–	–
Number of drugs following MTB	>1	52	5.03 (1.96–8.11)	1.12 (0.66–1.90)	0.675	–	–	9.80 (2.42–17.18)	1.12 (0.59–2.12)	0.725	–	–
	1	28	4.00 (1.49–6.51)	–	–	–	–	12.80 (1.88–23.72)	–	–	–	–
MSI-H	Yes	6	NR	0.16 (0.02–1.14)	0.067	0.19 (0.03–1.39)	0.102	NR	0.25 (0.03–1.83)	0.171	–	–
	No/unknown^a^	74	4.03 (2.28–5.79)	–	–	–	–	9.80 (4.91–14.70)	–	–	–	–
TMB (Muts/Mb)	≥10	21	9.30 (4.39–14.21)	0.66 (0.36–1.22)	0.183	–	–	NR	0.60 (0.27–1.35)	0.215	–	–
	<10/unknown^a^	59	3.47 (1.93–5.01)	–	–	–	–	8.30 (2.88–13.72)	–	–	–	–
TMB (Muts/Mb)	≥20	7	9.30 (0.00–19.88)	0.57 (0.18–1.82)	0.340			NR	0.75 (0.18–3.11)	0.687	–	–
	<20/unknown^a^	73	4.03 (2.22–5.85)					11.57 (6.09–17.04)	–	–	–	–
PDL1 IHC tumor (% TPS)	≥1	23	6.10 (0.00–13.93)	0.99 (0.57–1.71)	0.959	–	–	NR	0.96 (0.48–1.91)	0.906	–	–
	<1/unknown^a^	57	4.50 (2.88-6.12)	–	–	–	–	12.80 (6.65–18.95)	–	–	–	–

Abbreviations: *CI* confidence interval, *GI* gastrointestinal, *HR* hazard ratio, *IHC* immunohistochemistry, *MSI-H* microsatellite instability high, *MTB* Molecular Tumor Board, *NR* not reached, *OS* overall survival, *PFS* progression-free survival, *TMB* tumor mutational burden, *TPS* tumor proportion score.

^a^Immune markers were not tested in some cases and are categorized as unknown in these patients: MSI-H (*N* = 23), TMB (*N* = 16), PDL1 IHC (*N* = 16).

^b^Covariates with *p*-value < 0.1 by univariate analysis were included in multivariate analysis.

### High versus low degrees of matching of treatment to molecular alterations was associated with a higher rate of clinical benefit (SD ≥ 6 months/PR/CR)

**Fig. 3:**
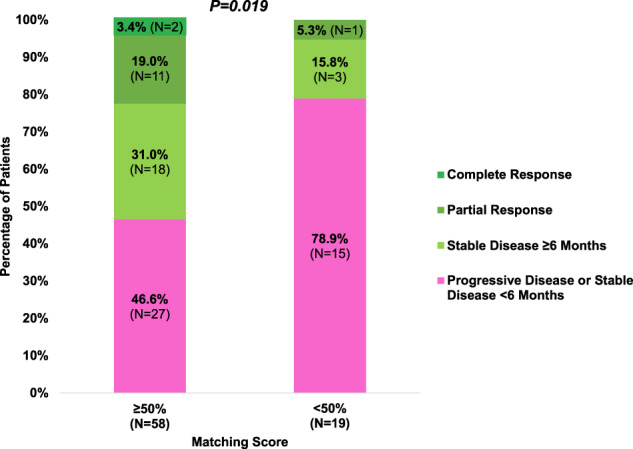
Clinical benefit rate (SD ≥ 6 months/PR/CR) based on Matching Score (*N* = 77*). Clinical benefit rate (SD ≥ 6 months/PR/CR) was significantly higher among patients who received therapy with high (≥50%) Matching Score (31/58 (53.4%)) when compared to patients with low (<50%) Matching Score (4/19 (21.1%)) (*p* = 0.019, univariate analysis). *Three of 80 patients were not evaluable for response since these patients had ongoing SD that was less than 6 months at the time of data cutoff. Abbreviations: CR complete response; PR partial response; SD stable disease.

We categorized patients who demonstrated stable disease (SD) ≥ 6 months, partial response (PR), or complete response (CR), as determined by physician assessment/RECIST criteria, as having clinical benefit (SD ≥ 6 months, PR, CR) from treatment^14,18^. In contrast, patients who had progressive disease (PD), or stable disease <6 months were categorized as not having clinical benefit from treatment. Consequently, patients with high degrees of matching of treatment to cognate alterations (i.e., high Matching Score (≥50%)) showed a significantly higher rate of clinical benefit (53.4% [31/58]) when compared to patients with lower degrees of matching (Matching Score (<50%) (21.1% [4/19]) (odds ratio [OR], 0.23; 95% CI, 0.07–0.79; *p* = 0.019; univariate analysis)) (Fig. 3). The association between degree of matching and clinical benefit rate maintained statistical significance with multivariate analysis (odds ratio [OR], 0.22; 95% CI, 0.06–0.77, *p* = 0.018) (Table 3).

**Table 3:** Association between patient and treatment characteristics and clinical benefit rate (SD ≥ 6 months/PR/CR) (*N* = 77^a^).

Characteristics		Clinical benefit rate (SD ≥ 6 months/PR/CR)					
				Univariate		Multivariate^c^	
		*N*	SD ≥ 6 months/PR/CR (*N*, %)	OR (95% CI)	*P*-value	OR (95% CI)	*P*-value
Age, years	≥63	39	23 (59%)	–	–	–	–
	<63	38	12 (32%)	0.32 (0.13–0.82)	0.017	0.30 (0.11–0.81)	0.017
Sex	Male	34	12 (35%)	–	–	–	–
	Female	43	23 (53%)	2.11 (0.84–5.31)	0.114	–	–
Matching score (%)	≥50%	58	31 (53%)	–	–	–	
	<50%	19	4 (21%)	0.23 (0.07–0.79)	0.019	0.22 (0.06–0.77)	0.018
GI malignancies	Yes	27	11 (41%)	–	–	–	–
	No	50	24 (48%)	1.34 (0.52–3.46)	0.542	–	–
Number of prior lines of therapy	≥3	33	16 (48%)	–	–	–	–
	<3	44	19 (43%)	0.81 (0.33–2.00)	0.644	–	–
Number of drugs following MTB	>1	49	23 (47%)	–	–	–	–
	1	28	12 (43%)	0.85 (0.33–2.16)	0.729	–	–
MSI-H	Yes	5	4 (80%)	–	–	–	–
	No/unknown^b^	72	31 (43%)	0.19 (0.02–1.78)	0.145	–	–
TMB (Muts/Mb)	≥10	20	12 (60%)	–	–	–	–
	<10/unknown^b^	57	23 (40%)	0.45 (0.16–1.28)	0.133	–	–
TMB (Muts/Mb)	≥20	6	4 (67%)				
	<20/unknown^b^	71	31 (44%)	0.39 (0.07–2.26)	0.291		
PDL1 IHC tumor (% TPS)	≥1	22	11 (50%)	–	–	–	–
	<1/unknown^b^	55	24 (44%)	0.77 (0.29–2.09)	0.613	–	–

Abbreviations: *CI* confidence interval, *CR* complete response, *GI* gastrointestinal, *IHC* immunohistochemistry, *MSI-H* microsatellite instability high, *MTB* Molecular Tumor Board, *OR* odds ratio, *PR* partial response, *TMB* tumor mutational burden, *TPS* tumor proportion score, *SD* stable disease.

^a^Three of 80 patients were not evaluable for response since these patients had ongoing SD that was less than 6 months at the time of data cutoff; hence it was too early for evaluation of this parameter.

^b^Immune markers were not tested in some cases and are categorized as unknown in these patients: MSI-H (N = 23), TMB (N = 16), PDL1 IHC (N = 16).

^c^Covariates with *p*-value < 0.1 by univariate analysis were included in multivariate analysis.

## Discussion

The Molecular Tumor Board (MTB) at the University of California San Diego Center for Personalized Cancer Therapy represents a real-world precision medicine experience demonstrating the use of innovative molecular profiling technologies to uniquely match cancer therapies to tumors. Several studies have demonstrated similar precision medicine strategies^19,20^. To facilitate MTB discussions, multiple clinical-laboratory tests were utilized, including tissue NGS, blood-derived cfDNA, mRNA, and IHC. In the case of cancer immunotherapy, the MTB considered multiple molecular correlates and immunotherapy biomarkers to evaluate the patients best suited for immune checkpoint blockade-based therapies. Furthermore, patients could receive matched targeted agents in addition to immune checkpoint blockade in order to enhance the degree of matching.

Ultimately, 80 patients with a wide range of advanced stage cancers, many of whom were refractory to multiple prior therapies (43% having received ≥3 prior lines of treatment), and with a range of difficult-to-treat malignancies, including gastrointestinal cancers/hepatobiliary/pancreatic cancer, were given a drug regimen that included ≥1 immune checkpoint inhibitor agent following MTB discussion. Sixty of these patients had tumors that were highly matched to therapy (Matching Score ≥50%) while 20 of them had lesser degrees of matching (Matching Score 0 to 49%).

Overall, patients with high versus low degrees of tumor-to-therapy matching based on multi-omic molecular profiles attained significantly longer PFS (*p* = 0.011) and OS (*p* = 0.014) and higher clinical benefit rates (SD ≥ 6 months/PR/CR (*p* = 0.019). No other factors, other than age, correlated significantly with outcome (Tables 2 and 3). Importantly, the use of combination therapy in and of itself was not a significant correlate of outcome (*p* > 0.6) indicating that it was not the number of drugs used, but rather the degree of matching of those drugs to the cancer molecular profile that was important.

Many studies have established the importance of individual biomarkers such as high TMB, MSI, and positive PDL1 IHC expression, as markers for predicting outcome to checkpoint blockade immunotherapy^8,21–32^. These findings have led to FDA-approved indications of immune-checkpoint inhibitors for tumors with elevated PD-L1 expression, MSI-H, and/or high TMB (≥10 muts/Mb) in various contexts^2,9,26–28^. However, these conclusions are largely based on a one-dimensional (single biomarker) approach to immunotherapy predictors. Our MTB did not evaluate biomarkers, whether for immunotherapy or for targeted therapies that were given in combination with immunotherapy, as stand-alone predictors, but rather incorporated multiple biomarkers, in order to assess the degree of matching (as reflected by the Matching Score—see “Methods”). Ultimately, what our study suggests is that the use of immunotherapy agents is best guided by a comprehensive review of tumor characteristics, and a multifactorial approach to developing treatment plans for patients.

Regarding dosing, we generally dosed ICBs at full dose and started concomitant drugs at half dose, monitoring the patient weekly and titrating to tolerance (unless the dosing of the combination had previously been established). Our prior studies^12^ have shown that this method of dosing is safe, with the number of drugs in the regimen being unrelated to the number of severe adverse events; furthermore, the severe adverse events deemed at least possibly drug-related trended lower in patients with a Matching Score ≥50% versus <50% (3.6% versus 15.6%; *p* = 0.14), perhaps because dosing was adjusted to individual tolerance. There were no treatment-related deaths in this study.

There are several important limitations to the present analysis. First, this study represents real-world data from the MTB at the University of California, San Diego, and is not a randomized controlled trial. Therefore, there may be unknown confounders. Second, this study was limited by a small sample size of 80 patients who presented to the face-to-face MTB, and subsequently were treated with immune checkpoint inhibitor agents and evaluable for treatment outcome. Third, it is unclear why some patients with higher matching scores did not respond; response to immune checkpoint blockade is multifactorial, and markers such as TMB and PDL1 are useful but imperfect predictors. Fourth, the number and distribution of cancer types represented in this study is limited by those presented at the MTB, potentially leading to selection bias. Finally, the heterogeneity of cancer types did not allow evaluation of outcomes in individual histologies, and might have confounded the data, though the presence of multiple cancer types might also suggest the possibility of generalizing these findings across the pan-cancer spectrum.

To summarize, our current observations demonstrate the utility of the MTB as a vehicle for implementing a precision medicine strategy to guide the use of cancer immunotherapy in treating advanced stage cancers. Utilizing multiple forms of biomarkers, including next generation sequencing, RNA expression and IHC, patients were evaluated and matched to immune checkpoint inhibitors with a multifactorial approach to treatment. Most patients received combination therapy, often including targeted agents matched to cognate molecular aberrations in addition to immunotherapy; however, combination therapy in and of itself was not predictive of outcome—rather it was the degree of matching that was important, suggesting that, in our cohort, simply adding agents without consideration for the underlying molecular landscape in individual malignancies was not sufficient to improve outcome. Overall, patients who were treated with regimens with a high versus low degree of matching of tumor characteristics to therapy exhibited improvement in all outcome parameters. Further prospective validation trials of this strategy are warranted.

## Methods

### Molecular Tumor Board

The Molecular Tumor Board (MTB) at the University of California San Diego Center for Personalized Cancer Therapy was composed of a multidisciplinary team of specialists including medical oncologists (pediatric and adult), radiation oncologists, surgeons including gynecologic oncologists, immunologists, pathologists, radiologists, geneticists, basic/translational scientists, bioinformaticians, and clinical trial coordinators/navigators. The MTB gathered approximately three times per month for face-to-face meetings to discuss the patients that were submitted by primary treating physicians. These meetings were led and moderated by a senior and a mid-level medical oncologist with expertize in genomics, immunotherapy, and clinical trials. Additionally, an MTB project manager prepared detailed meeting agendas with de-identified patient information, including age, sex, diagnosis, pathology, treatment history, and molecular profiling information, including specific test and service used, the date of specimen tested, and the molecular diagnostics report. All clinical laboratory tests used for MTB assessment were obtained from Clinical Laboratory Improvement Amendments (CLIA)-licensed and College of American Pathologists (CAP)-accredited institutions.

Collectively, all patient information, laboratory tests, imaging, and pathology were evaluated in MTB discussions. These discussions focused particularly on the presence of tumor molecular alterations, either somatic or germline, in various signaling pathways, and which drugs, either approved or in clinical trials, could potentially impact the molecular findings. To assist in this process, a medication acquisition specialist and clinical trial coordinator/navigator were present at the MTB meeting to facilitate the ordering of medications (either on- or off-label approved), screening for clinical trials, and obtaining written and informed consent. The MTB abided by all Health Insurance Portability and Accountability Act (HIPAA) privacy laws. An MTB physician facilitator as well as the presenting physician ensured the accuracy of all meeting notes and recommendations before inputting them into the medical record. All MTB recommendations were considered advisory, with treatment decisions to be made at the discretion of the primary treating physician.

### Patients and therapy

The patients in this study were drawn from a cohort of 715 patients who presented to the face-to-face MTB, and subsequently, 429 patients who were assessable for clinical therapeutic outcome following MTB discussion (Fig. 1)^14^. The most common reason for exclusion was that patients either did not receive treatment or their treatment did not change within six months after MTB presentation. From this subset, the current study evaluates in depth, the 80 patients with various types of cancer whose treatment regimen included immune checkpoint inhibitor(s) following MTB discussion. The attending physician made all treatment decisions and could choose to follow or not follow the MTB recommendations. Regarding dosing, immunotherapy was generally dosed at full dose, while other agents were started at most, at half dose, unless dosing had previously been established. Patients were initially seen weekly as needed and dosing was adjusted to tolerance. This method has been described previously^12^ and was based on extensive review of the literature of dosing regimens across approximately 80,000 patients in clinical trials^33–36^. Electronic medical records were utilized to obtain de-identified patient characteristics and treatment outcomes. This study followed the guidelines of the Institutional Review Board (IRB)-approved UCSD-Profile Related Evidence Determining Individualized Cancer Therapy (PREDICT) study (NCT02478931) and any additional investigational studies for which patients gave written informed consent.

### Molecular profiling

Next-generation sequencing (NGS) was performed on tissue and blood in one of several Clinical Laboratory Improvement Amendment (CLIA)-certified laboratories (Supplementary Table 1). Sequencing panels ranged from 182 to 596 genes for tissue and 54 to 74 for blood-derived cell-free circulating tumor DNA (cfDNA). Immune markers, including tumor mutational burden (TMB), microsatellite instability (MSI), and PD-L1 immunohistochemistry (IHC) were also evaluated in most patients (Supplementary Table 1). The attending physician chose all tests that were ordered.

In some cases, patients received the same immunotherapy biomarker test done by multiple laboratories. In this current study, the lab results included for analysis were based on the following method: for MSI and TMB testing, priority was given to results from the Foundation Medicine laboratory. The reasoning for prioritizing this laboratory is to reduce the amount of variability in MSI and TMB testing and reporting that exists between different laboratories. Furthermore, Foundation Medicine was prioritized specifically, because the majority of the patients in this study received testing from this laboratory, whereas only a small minority received exclusive testing from other laboratories (Supplementary Table 1). For PD-L1 immunohistochemistry (IHC) testing, if any laboratory reported positive expression (e.g., ≥1% tumor proportion score (TPS) or intermediate/high positive on RNA expression analysis (Omniseq))^37^, this was included in our analysis as positive.

### Statistical methods and endpoints

Patient characteristics and molecular profiling information were presented with descriptive statistics. The primary outcome variables were progression-free survival (PFS) and overall survival (OS). PFS is the time between the date of immune checkpoint blockade treatment onset after MTB presentation and the date of progression, as determined by clinical evaluation or imaging. OS is the time between the date of treatment onset after MTB presentation and the date of death or last follow-up. Patients were censored for PFS at last follow-up date if their tumor had not shown progression at that date. Patients were censored for OS if they were alive at last follow-up date. Clinical response was evaluated based on RECIST criteria^18^. Survival analysis was assessed using Kaplan–Meier analysis and Cox regression to compare subgroups of patients. Binomial logistic regression was used to compare clinical benefit rates between subgroups. *P*-values ≤ 0.05 were considered significant. Statistical analyses were performed with R and SPSS, version 25 (*IBM Corporation*, Armonk, NY).

### Matching score

Matching Score was a percentage assigned to each patient based on the number of molecular alterations that were targeted by drugs administered following MTB presentation. Matching Score was calculated via *post hoc* analysis by investigators that were blinded to treatment outcomes, based on drugs given. As previously described, the Matching Score calculation included evaluation of all NGS characterized variants (but not variants of unknown significance [VUS]) as well as mRNA expression, protein expression, and immunotherapy biomarkers in select cases^12^.

Briefly, the Matching Score was calculated by taking the number of alterations targeted by drugs given divided by the total number of alterations. Higher Matching Score equated to better matching between drugs and alterations (0%, unmatched, 100% completely matched). For example, if a tumor had 12 pathogenic genomic alterations and the patient received two drugs that targeted six of these alterations, the score would be 50% (6 of 12); if a patient had two pathogenic genomic alterations and received drugs that matched both of them, the score was 100%. In the case of immunotherapy, scoring also considered immune biomarkers. If a patient received immune checkpoint inhibitor therapy and their immune biomarker profile showed high microsatellite instability (MSI-H) (or other evidence of a mismatch repair gene defect), high tumor mutational burden (TMB) (defined at the time as ≥20 mutations/Mb on tissue sampling), and/or high-positive expression of PD-L1 by immunohistochemistry (IHC) (≥30% TPS), they were given a score of 100%. If a patient had intermediate TMB (defined at the time as 6–19 mutations/Mb) or low-positive PD-L1 by IHC (1–29% TPS) and received immunotherapy, they were given a score of 50% (the study design predated the FDA cut off of ≥10 mutations/megabase for high TMB). *PD-L1* amplification also garnered a 50% Matching Score. Furthermore, if a patient in the immunotherapy-treated group had, for example TMB intermediate (scored 50%) but also received matched targeted therapy, their score was determined by adding 50% (from TMB) to 0.5*([number of alterations targeted by drugs given]/[total number of alterations]) (e.g., if tumor had intermediate TMB and received a checkpoint inhibitor, but also had an *ROS1* and *KRAS* alteration and received a *ROS1* inhibitor in addition to checkpoint blockade, the score was 50% plus 0.5*(1/2) = 50% plus 25% = 75%). Additional details regarding Matching Score calculations have been previously reported^12,14^. Similar to previous studies, we stratified patients into high Matching Score (≥50%) and low Matching Score (<50%) groups^12,14^. Details on individual patient characteristics, molecular alterations, therapies given, and Matching Score are provided in Supplementary Table 3.

### Reporting summary

Further information on research design is available in the Nature Research Reporting Summary linked to this article.

## Supplementary information

Supplementary Information

Reporting Summary

## Data Availability

The specific molecular assays used by the Molecular Tumor Board included Next-generation sequencing (NGS) performed on tissue and blood, mRNA, immunohistochemistry (IHC), as well as specific biomarkers including tumor mutational burden (TMB), microsatellite instability (MSI), and PD-L1 IHC. All data were obtained from one of several Clinical Laboratory Improvement Amendment (CLIA)-certified laboratories (Supplementary Table 1). All datasets used and/or analyzed during the current study are available from the corresponding author upon reasonable request.
