# Supplementary material for: Pan-cancer molecular tumor board experience with biomarker-driven precision immunotherapy
Source: NPJ Precis Oncol. 2022 Sep 22;6:67. doi: 10.1038/s41698-022-00309-0 (PMC9500013; doi:10.1038/s41698-022-00309-0)
Supplement: Supplementary file 1 — Supplementary Information [file 41698_2022_309_MOESM1_ESM.pdf]

Supplementary Table 1. Molecular diagnostics tests and laboratory used for next generation sequencing and immunotherapy biomarkers (N=80 unique patients)

Molecular Diagnostics Tests	N, patients
**Tissue next-generation sequencing**	**75**
Foundation Medicine	72
Paradigm	2
Caris	1
**Cell-free circulating tumor DNA**	**36**
Guardant	31
Foundation Medicine	5
**Microsatellite Instability**	**57**
Foundation Medicine	54
Caris	1
OmniSeq	1
Response Genetics	1
**Tumor Mutational Burden**	**64**
Foundation Medicine	61
OmniSeq	2
Caris	1
**PD-L1 Immunohistochemistry**	**64**
Foundation Medicine	52
Caris	6
Paradigm	5
OmniSeq	1
**PD-L1 RNA**	**8**
Omniseq	8

*Websites of CLIA-certified testing laboratories:
Caris, https://www.carismolecularintelligence.com/molecular-testing-services/;
Foundation Medicine, https://www.foundationmedicine.com/;
Guardant, http://www.guardant360.com/;
OmniSeq, https://www.omniseq.com/;
Paradigm, https://www.paradigmdx.com/;
Response Genetics, https://www.vyantbio.com/;

Supplementary Table 2. Types of drugs administered to patients who received >1 drug after MTB discussion (N=52)

Study ID	Therapies given after MTB discussion	# of therapies	Immune checkpoint inhibitor	Targeted therapy	Second immunotherapy drug given	Chemotherapy	Hormonal therapy
1837	IDO inhibitor trial with pembrolizumab	2	pembrolizumab		IDO inhibitor trial		
1874	nivolumab, bevacizumab	2	nivolumab	bevacizumab			
2774	nivolumab, vismodegib, anastrozole	3	nivolumab	vismodegib			anastrazole
2779	cetuximab, erlotinib, nivolumab	3	nivolumab	cetuximab, erlotinib			
3043	trametinib, pembrolizumab	2	pembrolizumab	trametinib			
1732	nivolumab, bevacizumab	2	nivolumab	bevacizumab			
3162	anti-CD73 trial with durvalumab	2	durvalumab		anti-CD73 trial		
2612	anti-CD73 trial with durvalumab	2	durvalumab		anti-CD73 trial		
3332	olaparib, nivolumab	2	nivolumab	olaparib			
3102	nivolumab, cabozantinib	2	nivolumab	cabozantinib			
3175	nivolumab, anastrozole, sulindac	3	nivolumab	sulindac			anastrazole
3453	lenvatinib, nivolumab, palbociclib	3	nivolumab	palbociclib, lenvatinib			
3568	paclitaxel protein-bound, gemcitabine, nivolumab	3	nivolumab			gemcitabine, paclitaxel	
3606	paclitaxel protein-bound, pembrolizumab	2	pembrolizumab			paclitaxel	
3618	nivolumab, olaparib	2	nivolumab	olaparib			
3799	cobimetinib, pembrolizumb	2	pembrolizumab	cobimetinib			
3634	nivolumab, ibrutinib	2	nivolumab	ibrutinib			
3166	durvalumab and tremelimumab	2	durvalumab		tremelimumab		
2597	atezolizumab, cobimetinib	2	atezolizumab	cobimetinib			
3797	durvalumab with STAT3 inhibitor trial	2	durvalumab	STAT3 inhibitor trial			
3893	trametinib, nivolumab	2	nivolumab	trametinib			
3867	nivolumab, lenvatinib, palbociclib	3	nivolumab	palbociclib, lenvatinib			
4014	anti-CD73 trial with durvalumab	2	durvalumab		anti-CD73 trial		
4183	azacitidine, pembrolizumab	2	pembrolizumab			azacitidine	
4275	trametinib, nivolumab	2	nivolumab	trametinib			
4340	palbociclib, pembrolizumab	2	pembrolizumab	palbociclib			
3984	ipilimumab, nivolumab	2	nivolumab		ipilimumab		
3515	atezolizumab, trastuzumab	2	atezolizumab	trastuzumab			
4760	trametinib, pembrolizumab, pazopanib	3	pembrolizumab	trametinib, pazopanib			
4188	pembrolizumab, cisplatin, trametinib, sulindac	4	pembrolizumab	trametinib, sulindac		cisplatin	
2837	pembrolizumab, lenvatinib	2	pembrolizumab	lenvantinib			
3692	gemcitabine, paclitaxel protein-bound, nivolumab	3	nivolumab			gemcitabine, paclitaxel	
4222	ipilimumab, nivolumab	2	nivolumab		ipilimumab		
4912	nivolumab, lenvatinib, everolimus	3	nivolumab	lenvantinib, everolimus			

4914	ipilimumab, nivolumab	2	nivolumab		ipilimumab		
4486	ramucirumab, paclitaxel, trastuzumab, pembrolizumab	4	pembrolizumab	trastuzumab, ramucirumab,		paclitaxel	
3026	nivolumab, trametinib, anastrozole, afatinib	4	nivolumab	trametinib, afatinib			anastrazole
3571	pembrolizumab, trastuzumab	2	pembrolizumab	trastuzumab			
5079	ibrutinib, pembrolizumab	2	pembrolizumab	ibrutinib			
3374	nivolumab, bevacizumab, palbociclib	3	nivolumab	bevacizumab, palbociclib			
4411	brentuximab, nivolumab	2	nivolumab	brentuximab			
5084	cabozantinib, nivolumab	2	nivolumab	cabozantinib			
4455	brentuximab, pembrolizumab	2	pembrolizumab	brentuximab			
4778	brentuximab, pembrolizumab	2	pembrolizumab	brentuximab			
3794	pembrolizumab, trastuzumab	2	pembrolizumab	trastuzumab			
4504	nivolumab, palbociclib, everolimus	3	nivolumab	palbociclib, everolimus			
4926	nivolumab, trametinib, olaparib	3	nivolumab	trametinib, olaparib			
4946	pembrolizumab, venetoclax	2	pembrolizumab	venetoclax			
5490	selective PI3Kγ Inhibitor, nivolumab	2	nivolumab	selective PI3Kγ Inhibitor			
3351	brentuximab, pembrolizumab	2	pembrolizumab	brentuximab			
5465	pembrolizumab, trametinib	2	pembrolizumab	trametinib			
5696	cabozantinib, nivolumab	2	nivolumab	cabozantinib			

* Twenty-one of the 28 patients (75%) who received one drug (single immune checkpoint inhibitor) following the MTB discussion had high Matching Score (≥50%) and 39 of the 52 (75%) patients who received >1 drug had high Matching Score. Of the 52 patients who received >1 drug, 40/52 (77%) received targeted therapy, 8/52 (15%) received a second immunotherapy, 3/52 (6%) received a hormonal agent, and 6/52 (12%) received chemotherapy as part of their regimen. Some patients received more than 2 drugs.

Supplementary Table 3. Clinical characteristics, molecular alterations, and therapies for all patients treated with immune checkpoint inhibitors after MTB presentation (N=80)

#	Study ID	Diagnostic category	Matching Score ≥50%	PFS (months)	Progression	OS (months)	Death	Clinical benefit	Therapy given after MTB	Pathogenic Molecular Alteration(s)	MSI-High	Tumor Mutational Burden	PDL1 IHC
			Yes=1, No=0		Yes=1, No=0		Yes=1, No=0	1: CR<br>2: PR<br>3: SD>=6mo<br>4: PD<br>5: PD (SD less than 6 months)<br>9: SD<6mo				Low: ≤5 muts/Mb<br>Intermediate: 6-19 muts/Mb<br>High: ≥20 muts/Mb<br>Unknown	Negative: 0% TPS<br>Low Positive: 1-29% TPS<br>High Positive: ≥30% TPS<br>Unknown
1	1195	Other malignancies	0	1	1	13	1	5	nivolumab	CCND1 amplification, ERBB2 amplification – equivocal, MET amplification – equivocal, CDKN2A/B loss, IGF1R amplification, MITF amplification – equivocal, MYC amplification, NOTCH1 V1676I, MUTYH G382D, RPTOR amplification	No	Unknown	Unknown
2	1837	Head and Neck cancer	0	3	1	3	1	5	IDO inhibitor trial with pembrolizumab	PIK3CA E545K, PIK3CA AMP, TP53 V216M, TP53 R273L, TP53 R181P, PDGFRA R804Q	No	Unknown	Unknown
3	1628	Head and Neck cancer	1	8	1	17	1	3	nivolumab	BRCA2 M965fs*13, S884*, ERBB2 I767M – subclonal, FGFR3 S249C, CDKN2A/B loss, TP53 R175H, BCORL1 Q851*, MLL3 Q2197* – subclonal, SMAD4 D351H. TP53 R175H, TP53 A161T, TP53 H168R , PDGFRA I472I , ATM R3008C, SMAD4 D351H, BRCA2 L901V, BRCA2 S884*, APC E761Q, FGFR3 L419L , FGFR3 *807S, FGFR3 S408Y, FBXW7 D643N, VHL E186K.	No	Unknown	Low positive
4	2083	Colorectal cancer	1	19	0	33	0	2	pembrolizumab	loss of expression of MSH6	Yes	Unknown	Unknown
5	2131	Colorectal cancer	1	2	1	4	1	5	nivolumab	KRAS wildtype, BRAF wildtype, CCND1 amplification, EGFR amplification, ERBB2 amplification, CDK6 amplification, FGF19 amplification, FGF4 amplification, MYC amplification, TOP2A amplification, FGF3 amplification, MUTYH Y165C, TP53 splice site 672+1G>T	No	Intermediate	Unknown
6	1874	CNS malignancies	0	7	1	9	1	3	nivolumab, bevacizumab	EGFR amplification, EGFRvIVa, CDKN2A loss p16INK4a and p14ARF exons 2-3, PIK3R1 N453del, QKI E135fs*5, SETD2 splice site 5016-2_5018delAGAAA, TERT promoter -124C>T, PDGFRA V547V	No	Unknown	Negative
7	2774	Gynecologic cancer	1	8	0	8	0	3	nivolumab, vismodegib, anastrozole	KRAS Q61H, PIK3CA P449T, R88Q, PTCH1 S1203fs*52, ARID1A G95fs*10, DNMT3A Q110fs*52, FUBP1 splice site 637-1G>A, NFE2L2 D13G, SOX9 E50*, N96fs*156, TP53 Y126C	Yes	Intermediate	Negative
8	2730	Biliary cancer	1	3	1	3	1	5	pembrolizumab	FLT4 R1223H, IDH1 R132L, BAP1 W52*, CDKN2A/B loss, SETD2 splice site 4918-2A>C	No	Low	Low Positive
9	2779	Gastroesophageal cancer	1	19	1	19	1	2	cetuximab, erlotinib, nivolumab	EGFR amplification, EGFR R224C	No	unknown	Unknown
10	3043	Sarcoma	0	5	1	18	1	5	trametinib, pembrolizumab	NF1 loss exons 54-57, RB1 truncation exon 1 and truncation exon 17, SUZ12 loss exons 4-16, TCF3 T168M	No	Low	Unknown
11	3000	Other malignancies	0	2	1	2	1	5	pembrolizumab	NF1 loss exons 16-57, NF2 Y177fs*1, TP53 F270S, ESR1 amplification	No	Low	Negative
12	3261	Colorectal cancer	1	24	0	24	0	2	pembrolizumab	AKT1 E17K, CYLD Q26* – subclonal, LRP1B T2117fs*5, SPTA1 R374*, STAG2 Q167*	No	Intermediate	Negative
13	1793	Colorectal cancer	0	2	1	12	1	5	pembrolizumab	FLT4 G71R, NRAS G12V, BRAF D594G, CTNNB1 K335I, T257I, MYC amplification, TERT promoter -124C>T, TP53 R175H	No	Low	Negative

14	3214	Gastroesophageal cancer	1	4	1	6	1	5	pembrolizumab	ABL1 NUP214-ABL1 fusion. CCNE1 amplification, NOTCH1 R365C – subclonal, TP53 R248W, ERBB2 V777L, KRAS Q61H, PIK3CA K111_I112insEK, TP53 I195T – equivocal, R248W	No	Intermediate	Negative
15	1732	CNS malignancies	1	2	1	5	1	5	nivolumab, bevacizumab	PIK3CA Q546L, MTOR S1641F, CDK4 amplification, PIK3CA Q546L, MDM2 amplification, FRS2 amplification, GLI1 amplification, TERT promoter -124C>T	No	Intermediate	Low Positive
16	3162	Pancreatic cancer	1	24	0	24	0	3	anti-CD73 trial with durvalumab	ERBB3 G284R, FBXW7 R505C, KRAS G12V, TP53 R273C, CDKN2A p16INK4a R58* and p14ARF P94L, p16INK4a R80* and p14ARF P72L, DNMT3A G543A, MSH6 R841fs*3, SMARCA4 R1135W	No	Unknown	High Positive
17	3285	Other malignancies	1	1	1	8	1	5	nivolumab	CCND3 amplification, CDKN2A/B loss, IGF1R amplification, TP53 loss exons 2-9, VEGFA amplification	No	Intermediate	Negative
18	2612	Colorectal cancer	0	2	0	4	1	9	anti-CD73 trial with durvalumab	KRAS G12S, APC E1353fs*21, SMAD4 R496H, TP53 C176Y	No	Low	Negative
19	3332	Gynecologic cancer	1	11	1	12	0	2	olaparib, nivolumab	ATM R982fs*15, BRCA2 G267*, PIK3CA amplification – equivocal, TP53 R342*, BCORL1 V676fs*51, CREBBP R75*, MAGI2 splice site 3031+1G>C, PRKCI amplification, TERC amplification. BRCA2 c.799G>T (G267X), TP53 c.1024C>T (R342X), NF1 Copy Number Loss , NOTCH1 c.7082A>T (Q2361L), PIK3R1 Copy Number Loss.	No	Intermediate	Negative
20	3102	CNS malignancies	1	6	1	15	1	3	nivolumab, cabozantinib	CD274 (PD-L1) amplification, CDK4 amplification – equivocal, KDR amplification, KIT amplification, MET amplification, PDCD1LG2 (PD-L2) amplification, PDGFRA amplification, MDM2 amplification – equivocal, CDKN2A/B loss, FRS2 amplification – equivocal, JAK2 amplification, RB1 splice site 2107-1G>C.	No	Low	Negative
21	3284	Gastroesophageal cancer	1	24	0	24	0	2	pembrolizumab	RNF43 loss exons 3-6, CDKN2A p16INK4a L78fs*41 and p14ARF H93fs*67, CTNNA1 loss, TP53 R248Q, CDKN2A p16INK4a L78fs*41 and p14ARF H93fs*67, TP53 R248Q	No	Intermediate	Negative
22	3357	Breast cancer	0	2	1	2	1	5	pembrolizumab	TP53 I251S, RET I852M, MET AMP, BRAF AMP	No	Unknown	Negative
23	3397	Gastroesophageal cancer	0	3	1	3	1	5	pembrolizumab	CCND1 amplification, FBXW7 R505G, KRAS A146V, PIK3CA amplification – equivocal, SOX2 amplification – equivocal, ARID1A W1073fs*32, FGF19 amplification, FGF3 amplification, FGF4 amplification, NFE2L2 D29G, NOTCH1 C423F, TP53 C277F	No	Low	Unknown
24	3412	Other malignancies	1	1	1	3	1	5	pembrolizumab	STK11 loss exons 1-6, CHD2 truncation exon 31, CCNE1 AMP	No	Intermediate	Low Positive
25	3414	Other GI cancer	1	2	1	4	1	5	pembrolizumab	KRAS L19F, PTEN G132V, MEN1 E466fs*70, ARID1A Q372fs*19, BCORL1 P1681fs*20, GNAS R201H, LRP1B M482I – subclonal, MSH2 Q288*, RB1 V654fs*4, SMAD4 R361H, TP53 Q167	Yes	Intermediate	Negative
26	3526	Sarcoma	0	14	0	14	0	3	nivolumab	KRAS Q61H, TP53 R273C	No	Unknown	Negative
27	3175	Hepatocellular Carcinoma	1	2	0	2	0	9	nivolumab , anastrozole, sulindac	CTNNB1 S33P, ARID1A Q548fs*71, TERT promoter -124C>T	No	Intermediate	Negative

28	3453	Gastroesophageal cancer	1	1	1	3	0	5	lenvatinib, nivolumab, palbociclib	CCND1 amplification, MET amplification, CDK6 amplification, ARID1A R1276*, FGF19 amplification, FGF3 amplification, FGF4 amplification, TERC amplification – equivocal, TP53 P278L, MGMT 0, 100 negative, RRM1 2, 40 negative, TOP2A 2, 25 positive, TOPO1 2, 90 positive	No	Intermediate	Low Positive
29	3568	Other malignancies	1	7	1	8	1	3	paclitaxel protein-bound, gemcitabine, nivolumab	CCNE1 amplification, RUNX1 E456*, SMAD4 truncation intron 5, TET2 P1962L, TP53 R213*, ER 2, 60 positive, ERCC1 2, 2 negative, RRM1 2, 10 negative, TOP2A 2, 20 positive, TOPO1 2, 70 positive, TUBB3 2, 20 negative	No	Intermediate	Negative
30	3543	Sarcoma	0	2	1	24	1	5	nivolumab	TLL2 G465R	No	Unknown	Negative
31	3606	Breast cancer	0	3	1	6	1	5	paclitaxel protein-bound, pembrolizumab	PTEN loss, MYC amplification, CCNE1 amplification, TP53 C275F	No	Low	Negative
32	3618	Hepatocellular Carcinoma	1	7	0	13	0	3	nivolumab, olaparib	APC G1203*, FANCL rearrangement intron 2, TERT promoter -124C>T, TP53 T125M, APC G1203*, CTNNB1 S33C	No	Intermediate	Negative
33	3799	Colorectal cancer	1	1	1	1	1	5	cobimetinib, pembrolizumb	NRAS Q61H – subclonal , Q61K – subclonal, JAK3 R403H, APC E1374*, ASXL1 truncation intron 6, BCL2L1 amplification, GNAS amplification, MUTYH G382D, SMAD2 R427*, SPTA1 R268*, SRC amplification, TOP1 amplification, TP53 P250S, R249S, hENT1 3, 80 positive, MET 1, 80 positive, PTEN 1, 1-4 negative	No	Intermediate	Negative
34	3634	Hematologic malignancies	1	21	0	21	0	2	nivolumab, ibrutinib	BRAF F595L, K601E – subclonal, KRAS A146V, BCOR L279fs*13, POT1 splice site 1594+1G>T, SETD2 M763fs*14	No	Low	Low Positive
35	3166	Colorectal cancer	0	2	1	2	1	5	durvalumab and tremelimumab	TP53 Y163fs*7, FBXW7 R367*, TSC2 duplication exons 17-31, APC E1286*, R554*, MAP2K4 loss	No	Low	Negative
36	2597	Colorectal cancer	0	3	1	3	1	5	atezolizumab, cobimetinib	ERCC1 2, 20 negative, KRAS G12V, APC Q1406fs*6, R805*, CDKN2A p14ARF S73R, SMAD4 loss, SOX9 Q230*, TP53 splice site 783-1G>T. APC R805*, KRAS G12V, MYC AMP	No	Low	Negative
37	3797	Hematologic malignancies	1	1	1	1	1	5	durvalumab with STAT3 inhibitor trial	DNMT3A P777fs*2, MAP2K1 (MEK1) P124R, TET2 T229fs*25, AR A404V, BCL7A splice site 92+1G>C, FOXP1 R514C, SOCS1 M1T	No	High	High Positive
38	3893	Hematologic malignancies	1	6	1	7		3	trametinib, nivolumab	NRAS Q61R, PTEN Q17*, CREBBP R768*, EP300 R1529*, SETD2 loss exons 19-21	No	Intermediate	Low Positive
39	3867	Gastroesophageal cancer	1	3	1	3	1	5	nivolumab, lenvatinib, palbociclib	CCND1 amplification, PIK3CA amplification, CDK6 amplification – equivocal, SOX2 amplification, CDKN2A/B loss, FGF19 amplification, FGF3 amplification, FGF4 amplification, PIK3CG amplification, PRKCI amplification, TERC amplification, TP53 G245D, MGMT 0, 100 negative, PTEN 1, 50 negative, RRM1 2, 10 negative, TOP2A 2, 10 positive, TS 0, 100 negative	No	Intermediate	Low Positive
40	4014	Pancreatic cancer	1	2	1	2	0	5	anti-CD73 trial with durvalumab	CKD4 amplification , ATM R2849 , TP53 H179D , GLI1 amplification , SMAD4 S232fs*3	No	Intermediate	Unknown
41	4183	Colorectal cancer	0	3	1	4	1	5	azacitidine, pembrolizumab	KRAS G12V, SMAD4 G419R	No	Unknown	Unknown
42	3892	Biliary cancer	1	17	1	22	0	3	atezolizumab	BRCA2 S1982fs*22, FBXW7 L700fs*3, AXIN1 R533_H534insQVHH, BCORL1 splice site 4531_4618+102del190, EP300 E1492*, KEAP1 splice site 1532-2A>T	No	Intermediate	High Positive

43	4187	Breast cancer	1	2	1	2	1	5	atezolizumab	PIK3CA H1047R, N345I, BRCA2 W3106*, CDH1 S9*, splice site 1320+1G>A, MUTYH rearrangement intron 1, PMS2 E531*, SMAD3 V331I, TP53 C176Y	No	High	Negative
44	4275	Gynecologic cancer	1	19	0	19	0	2	trametinib, nivolumab	NF1 R440*, TP53 T231fs*9	No	Unknown	Negative
45	4340	Pancreatic cancer	0	1	1	1	1	5	palbociclib, pembrolizumab	KRAS G12D, CDK6 amplification, CDKN2A/B loss, KDM6A V326fs*38, TP53 S127F, ZNF703 amplification	No	Low	Unknown
46	3984	Head and Neck cancer	1	5	1	16	0	5	Ipilimumab, nivolumab	BRCA2 V1238fs*21, PTEN loss exon 2, ATRX splice site 6803_6849+36del83, FAT1 P3967fs*73, SMAD4 E538* – subclonal, TP53 P75fs*48	No	Intermediate	Negative
47	3644	Gastroesophageal cancer	1	2	1	5	0	5	pembrolizumab	CCND1 amplification, FLT3 amplification, KRAS amplification, CDK6 amplification, MYC amplification, CCND3 amplification, CDK8 amplification, FGF19 amplification, FGF3 amplification, FGF4 amplification, GATA6 amplification – equivocal, TP53 R282W, VEGFA amplification	No	Intermediate	Negative
48	3515	Bladder/Ureter cancer	1	3	1	5	0	5	atezolizumab, trastuzumab	ATM Q1627*, ERBB2 amplification, CREBBP S575fs*16, RB1 E315*, TERT promoter -124C>T	No	Intermediate	Negative
49	4760	Colorectal cancer	1	11	1	12	0	3	trametinib, pembrolizumab, pazopanib	TP53 S241Y, KRAS G12C, BTLA moderate co-inhibitory, CTLA4 moderate co-inhibitory	No	Intermediate	Negative
50	4188	Biliary cancer	1	15	1	15	0	2	pembrolizumab, cisplatin, trametinib, sulindac	BRAF Exon 15 D594G, CTNNB1 Exon 3 S45F, TP53 Exon 8 D281fs, CCND3 amplified, ERCC1 IHC negative, 2+, 30%, Her2/Neu IHC negative, 1+, 3%, TOPO1 IHC positive, 2+, 40%, BRAF D594G, RAF1 AMP	No	Intermediate	Negative
51	2837	Sarcoma	1	13	0	13	0	3	pembrolizumab, lenvatinib	KIT K558_E562del, N822K, V654A, ARID1A truncation exon 18, NOTCH2 P6fs*27, FGFR1 AMP, MYC AMP, ERBB2 (HER2) AMP	No	Intermediate	Unknown
52	3692	Pancreatic cancer	1	9	1	14	0	3	gemcitabine, paclitaxel protein-bound, nivolumab	KRAS G12D, CCNE1 amplification, RB1 E282*, TP53 R273C	No	Intermediate	Negative
53	4222	Gynecologic cancer	1	1	1	16	0	5	ipilimumab, nivolumab	BLM N454fs*17, KMT2C (MLL3) E1689fs*28, TERT promoter -124C>T	No	Unknown	Low Positive
54	4936	Gynecologic cancer	1	9	1	13	0	3	pembrolizumab	ERBB2 V842I, FBXW7 R689W, PTEN R130Q, R233*, ABL1 R712H, ARID1A D1850fs*4, CHD4 R347*, DNMT3A Y533C – subclonal, FOXP1 R514H, MAP3K1 G234*, MLL2 R1903*, MSH6 L449fs*6, PIK3R1 splice site 1425+1G>A, PPP2R1A R183W, SMARCA4 splice site 3547-1G>T, TP53 R306*, R337C	No	High	Low Positive
55	4912	Other malignancies	1	7	0	7	0	3	nivolumab, lenvatinib, everolimus	RET NCOA4-RET fusion, FBXW7 R465C, RNF43 G659fs*41, APC S1272fs*4, T1556fs*3, ASXL1 R596fs*23, BCOR P1587fs*53, BCORL1 P1681fs*20, FANCC K231del, FAT1 F402fs*12, FLCN H429fs*39, MLL2 P647fs*283, Q827fs*3, RANBP2 L811R – subclonal, SMARCA4 R741fs*82, splice site 1761+2T>G, SOX9 G263fs*16, SPEN R2332H	Yes	High	Low Positive
56	4914	Gynecologic cancer	1	14	1	14	0	3	ipilimumab, nivolumab	ATM splice site 6096-1G>C, CTNNB1 T297fs*22, LRP1B deletion exons 4-14, NFKBIA amplification, NKX2-1 amplification – equivocal, SDHA deletion exons 6-7, TP53 R175H, ZNF217 amplification – equivocal	No	Intermediate	Negative
57	4486	Gastroesophageal cancer	1	3	1	4	1	5	ramucirumab, paclitaxel, trastuzumab, pembrolizumab	ERBB2 amplification – equivocal, NOTCH1 V1575L – subclonal, TP53 R248W, FBXW7 R465C, TOP2A 1, 20 positive, TOPO1 2, 100 positive, TUBB3 2, 15 negative, ERCC1 2, 35 negative, TS 0, 100 negative	No	Low	High Positive

58	3026	Pancreatic cancer	1	3	1	3	0	5	nivolumab, trametinib, anastrozole, afatinib	CDKN2A_p R80*, KRAS G12D, SMARCB1 R201Q, NF2 I126S, ERBB2 R816H	No	Intermediate	Negative
59	3571	Bladder/Ureter cancer	1	5	1	10	1	5	pembrolizumab, trastuzumab	ALK amplification, ERBB2 amplification, ERBB3 amplification, MYCN amplification, CCNE1 amplification – equivocal, CDKN2A/B loss, KDM6A Q240*, MCL1 amplification, TERT promoter -124C>T, TP53 Q192*	No	Intermediate	Negative
60	5079	Gastroesophageal cancer	0	13	1	13	0	2	ibrutinib, pembrolizumab	FBXW7 splice site 502-1_507delGATGAAA, IDH1 R132C, RNF43 truncation intron 5, ARID1A Q1519fs*13, MYD88 S219, PARK2 T240M, TP53 splice site 919+1G>C, ERCC1 negative, TUBB3 negative, TOP2A 2+ TOPO1 2+, TS negative, MYD88 S219C 10.83, IL7R R267fs 8.70, ERCC2 R487L 8.00, CUX1 c.*2200delT 11.48%, AREG copy loss, IFNA6 copy loss, RHOA Y42C	No	Unknown	Unknown
61	3374	Gastroesophageal cancer	1	8	1	8	1	1	nivolumab, bevacizumab, palbociclib	TSC2 splice site 2967-2A>T, MYC amplification, RICTOR amplification, CCND3 amplification, CTCF rearrangement exon 11, MYST3 amplification – equivocal, TP53 R175H, VEGFA amplification	No	Low	Low Positive
62	4411	Hematologic malignancies	1	2	1	2	1	5	brentuximab, nivolumab	CDKN2A/B loss, FAS G238*, TNFAIP3 K287fs*8, V273fs*5	No	Intermediate	High Positive
63	3896	CNS malignancies	1	5	1	8	1	5	nivolumab	CD274 (PD-L1) amplification – equivocal, FGFR1 N546K, NF1 C1016*, PIK3CA amplification – equivocal , E545K, SOX2 amplification – equivocal, ATRX E361fs*1, CDKN2A/B loss, JAK2 amplification – equivocal	No	Low	Unknown
64	5084	Gynecologic cancer	0	3	1	5	1	5	cabozantinib, nivolumab	HRAS amplification, PIK3CA E545K, AKT2 amplification, AXL amplification, MYC amplification, CCNE1 amplification, IGF2 amplification, PPP2R1A W257C, TP53 R306*	No	Low	Negative
65	5304	Gastroesophageal cancer	1	10	0	4	0	3	pembrolizumab	PIK3CA Y1021C, RICTOR amplification, BCOR Q312*, FGF10 amplification , MYST3 S1496L, PIK3CG R839C, TET2 R550*, TP53 R175H, R273C	No	Intermediate	Unknown
66	4455	Hematologic malignancies	1	2	1	4	1	5	brentuximab, pembrolizumab	IGH IGH-MYC rearrangement, STAT3 D661Y, BCOR loss exons 2-4, SGK1 Q167H	No	Intermediate	High Positive
67	4778	Hematologic malignancies	1	7	1	13	1	2	brentuximab, pembrolizumab	B2M E97*, CBL C396Y, CD58 S87fs*2, NFKBIA L20fs*25, splice site 179_227+119del168, SOCS1 A3T, D63fs*47, L147fs*9, PDCD1LG2 rearrangement	No	Intermediate	Negative
68	3794	Bladder/Ureter cancer	1	6	1	11	0	3	pembrolizumab, trastuzumab	ERBB2 S310F, ERBB3 E150K, HRAS Q61L, STAG2 R1207fs*6, TERT promoter -124C>T	No	Intermediate	Negative
69	4504	Gynecologic cancer	1	2	1	2	1	5	nivolumab, palbociclib, everolimus	PIK3CA E545K, CDKN2A p16INK4a deletion and p14ARF deletion exons 2-3, p16INK4a R58* and p14ARF P72L, FAT1 S3005*, TERT promoter -124C>T, TP53 E286K	No	Intermediate	Negative
70	5281	Breast cancer	1	10	0	10	0	1	pembrolizumab	BRCA2 N243fs*2, PIK3CA C604R, ESR1 D538G, APC R2237*, ARID1A G779*, CDKN2A p16INK4a W110* and p14ARF G125R, CIC splice site 1360+1G>A, CUL3 C251*, FAS C135fs*52, MLH1 splice site 678-1G>T, MLL2 P2354fs*30, NOTCH3 G2035fs*60, SMAD4 S32fs*1 – subclonal, TP53 R273H	Yes	High	Negative

71	4926	Other malignancies	1	3	0	3	0	9	nivolumab, trametinib, olaparib	ATM K2237fs*20, BRCA2 T3033fs*29, CCND1 V293G, ERBB3 N126K, FGFR3 V677I, KRAS G12A, PIK3CA G106R, R93W, PTEN R130P,RNF43 G659fs*41, ARID1A P2005fs*10, ARID1B splice site 2513-2A>G, CTCF T204fs*26, CTNNA1 R546*, EZH2 E225fs*1, FUBP1 S401fs*2, JAK1 P430fs*2, SMAD4 S32fs*1	Yes	High	Low Positive
72	4946	Hematologic malignancies	1	1	1	2	1	5	pembrolizumab, venetoclax	IGH IGH-BCL2 rearrangement, BCL2 G47D, CREBBP K663*, DDX3X R311fs*10, FOXO1 T24I, RB1 loss exons 1-12, TP53 R273C	No	Intermediate	High Positive
73	5490	Head and Neck cancer	0	8	1	10	0	3	selective PI3Kγ Inhibitor; nivolumab	EGFR exon 20 insertion (D770_N771insSVD), FGFR1 amplification, PIK3CA amplification, PIK3CB amplification, SOX2 amplification, DICER1 S665*, EPHB1 amplification, FAT1 N1864fs*2, Q1244*, MYST3 amplification, PRKCI amplification, TERC amplification, TP53 splice site 559+1G>A	No	Low	Unknown
74	3351	Hematologic malignancies	1	9	0	9	0	2	brentuximab, pembrolizumab	CD274 (PD-L1) amplification – equivocal, JAK2 amplification – equivocal, RICTOR amplification, ARID1A G149fs*77, FGF10 amplification, KDM4C amplification – equivocal, SOCS1 S116N, TNFAIP3 loss exons 3-4, TP53 G266R	No	Intermediate	High Positive
75	5467	Gynecologic cancer	1	11	1	12	0	3	pembrolizumab	CD274 (PD-L1) amplification – equivocal, JAK2 amplification – equivocal	No	Unknown	High Positive
76	5448	Sarcoma	1	14	1	17	0	2	atezolizumab	CCND1 amplification, CD274 (PD-L1) amplification, PDCD1LG2 (PD-L2) amplification, PDGFRB amplification – equivocal, BIRC3 amplification, CCNE1 amplification – equivocal, CDKN2A/B loss, CRKL amplification, CSF3R amplification – equivocal, FGF10 amplification, FGF19 amplification, FGF3 amplification, FGF4 amplification, JAK2 amplification, JUN amplification, KDM4C amplification, MAP3K14 amplification, MYCL1 amplification – equivocal	No	Intermediate	Low Positive
77	5465	Other malignancies	1	3	1	8	1	5	pembrolizumab, trametinib	NOTCH1 L2434fs*1, PIK3R1 N441_L449del, SPEN I1052fs*7, TP53 R273H, BRAF Amp	No	Intermediate	Negative
78	5480	Gynecologic cancer	1	4	1	7	1	5	pembrolizumab	FGFR1 amplification – equivocal, PIK3CA R88Q, PTEN S287*, MYCN amplification, FGF12 amplification – equivocal, TP53 V147fs*23, WHSC1L1 amplification – equivocal, ZNF703 amplification - equivocal	No	Intermediate	Negative
79	5637	Gastroesophageal cancer	1	8	0	8	0	3	pembrolizumab	BRCA2 splice site 8332-1G>A, KRAS G13D, ARID1A Y551fs*68, CASP8 F373fs*26, CDH1 P744fs*26, EP300 H368fs*5, GNAS R201C, MLL2 R2471*, MSH2 Q264fs*10, loss exons 1-7, RB1 R320*, K192fs*10, V654M, SMAD4 R497H, TP53M243I	No	High	Unknown
80	5696	Gynecologic cancer	0	4	1	8	0	5	cabozantinib, nivolumab	FGFR1 amplification, PIK3CA H1047R, NSD3 (WHSC1L1) amplification, TP53 V122fs*26	No	Low	Negative
