# Supplementary material for: Pan-cancer molecular tumor board experience with biomarker-driven precision immunotherapy
Source: NPJ Precis Oncol. 2022 Sep 22;6:67. doi: 10.1038/s41698-022-00309-0 (PMC9500013; doi:10.1038/s41698-022-00309-0)
Supplement: Supplementary file 2 — Reporting Summary [file 41698_2022_309_MOESM2_ESM.pdf]

nature portfolio

Corresponding author(s): Bryan Louie and Shumei Kato

Last updated by author(s): Aug 22, 2022

Reporting Summary

Nature Portfolio wishes to improve the reproducibility of the work that we publish. This form provides structure for consistency and transparency in reporting. For further information on Nature Portfolio policies, see our Editorial Policies and the Editorial Policy Checklist.

Statistics

For all statistical analyses, confirm that the following items are present in the figure legend, table legend, main text, or Methods section.

n/a	Confirmed	
☐	☒	The exact sample size (*n*) for each experimental group/condition, given as a discrete number and unit of measurement
☒	☐	A statement on whether measurements were taken from distinct samples or whether the same sample was measured repeatedly
☐	☒	The statistical test(s) used AND whether they are one- or two-sided<br>*Only common tests should be described solely by name; describe more complex techniques in the Methods section.*
☐	☒	A description of all covariates tested
☐	☒	A description of any assumptions or corrections, such as tests of normality and adjustment for multiple comparisons
☐	☒	A full description of the statistical parameters including central tendency (e.g. means) or other basic estimates (e.g. regression coefficient) AND variation (e.g. standard deviation) or associated estimates of uncertainty (e.g. confidence intervals)
☐	☒	For null hypothesis testing, the test statistic (e.g. *F*, *t*, *r*) with confidence intervals, effect sizes, degrees of freedom and *P* value noted<br>*Give P values as exact values whenever suitable.*
☒	☐	For Bayesian analysis, information on the choice of priors and Markov chain Monte Carlo settings
☒	☐	For hierarchical and complex designs, identification of the appropriate level for tests and full reporting of outcomes
☒	☐	Estimates of effect sizes (e.g. Cohen's *d*, Pearson's *r*), indicating how they were calculated

Our web collection on statistics for biologists contains articles on many of the points above.

Software and code

Policy information about availability of computer code

Data collection	No software was used
Data analysis	IBM SPSS Statistics Version 27; R Studio Build 461

For manuscripts utilizing custom algorithms or software that are central to the research but not yet described in published literature, software must be made available to editors and reviewers. We strongly encourage code deposition in a community repository (e.g. GitHub). See the Nature Portfolio guidelines for submitting code & software for further information.

Data

Policy information about availability of data

All manuscripts must include a data availability statement. This statement should provide the following information, where applicable:

- Accession codes, unique identifiers, or web links for publicly available datasets
- A description of any restrictions on data availability
- For clinical datasets or third party data, please ensure that the statement adheres to our policy

The specific molecular assays used by the Molecular Tumor Board included Next-generation sequencing (NGS) performed on tissue and blood, mRNA, immunohistochemistry (IHC), as well as specific biomarkers including tumor mutational burden (TMB), microsatellite instability (MSI), and PD-L1 IHC. All data were obtained from one of several Clinical Laboratory Improvement Amendment (CLIA)-certified laboratories (Supplementary Table 1). All datasets used and/or analyzed during the current study are available from the corresponding author upon reasonable request.

Human research participants

Policy information about studies involving human research participants and Sex and Gender in Research.

Reporting on sex and gender	Among the 80 patients in this study, biological sex was collected and reported. Forty-four patients (55%) were female and 36 patients (45%) were male. Sex was included as a covariate in multivariate analysis.
Population characteristics	Age, sex, number of prior therapies, presence of GI malignancy, MSI-H status, TMB status, PDL1 IHC status
Recruitment	Patients were recruited at UCSD Moores Cancer Center through clinic visits. The recruitment was based on physician's decision, which may have lead to selection bias.
Ethics oversight	This study was approved by UCSD Institutional Review Board. Informed consent was obtained from a legally acceptable representative.

Note that full information on the approval of the study protocol must also be provided in the manuscript.

Field-specific reporting

Please select the one below that is the best fit for your research. If you are not sure, read the appropriate sections before making your selection.

☒ Life sciences ☐ Behavioural & social sciences ☐ Ecological, evolutionary & environmental sciences

For a reference copy of the document with all sections, see nature.com/documents/nr-reporting-summary-flat.pdf

Life sciences study design

All studies must disclose on these points even when the disclosure is negative.

Sample size	Sample size was based on number of patients who presented to the face-to-face MTB during the specified time period. No sample size calculations were performed.
Data exclusions	The patients in this study were drawn from a cohort of 715 patients who presented to the face-to-face MTB, and subsequently 429 patients who were assessable for clinical therapeutic outcome following MTB discussion. The most common reason for exclusion was that patients either did not receive treatment or their treatment did not change within six months after MTB presentation. From this subset, the current study evaluates the 80 patients with various types of cancer whose treatment regimen included immune checkpoint inhibitor(s) following MTB discussion.
Replication	Replication was not feasible in this study. This is an observation study among patients who were presented at the Molecular Tumor Board and it is a unique patient population.
Randomization	This is an observation study that includes real-world data from the MTB at the University of California, San Diego, and is not a randomized controlled trial. Covariates were collected and reported as above and included in multivariate analysis.
Blinding	Blinding was not feasible since this is an observation study.

Reporting for specific materials, systems and methods

We require information from authors about some types of materials, experimental systems and methods used in many studies. Here, indicate whether each material, system or method listed is relevant to your study. If you are not sure if a list item applies to your research, read the appropriate section before selecting a response.

Materials & experimental systems

n/a	Involved in the study	
☒	☐	Antibodies
☒	☐	Eukaryotic cell lines
☒	☐	Palaeontology and archaeology
☒	☐	Animals and other organisms
☐	☒	Clinical data
☒	☐	Dual use research of concern

Methods

n/a	Involved in the study	
☒	☐	ChIP-seq
☒	☐	Flow cytometry
☒	☐	MRI-based neuroimaging

Clinical data

Policy information about clinical studies

All manuscripts should comply with the ICMJE guidelines for publication of clinical research and a completed CONSORT checklist must be included with all submissions.

Clinical trial registration	NCT02478931
Study protocol	https://clinicaltrials.gov/ct2/show/NCT02478931
Data collection	Data was collected at UCSD Moores Cancer Center
Outcomes	Clinical outcomes (progression-free survival, overall survival and clinical response) that were not predefined were assessed using logistic regression and Kaplan-Meier analysis. Due to the nature of this observation study, there are no predefined primary and secondary endpoints.
